# Reassortment between Avian H5N1 and Human Influenza Viruses Is Mainly Restricted to the Matrix and Neuraminidase Gene Segments

**DOI:** 10.1371/journal.pone.0059889

**Published:** 2013-03-20

**Authors:** Eefje J. A. Schrauwen, Theo M. Bestebroer, Guus F. Rimmelzwaan, Albert D. M. E. Osterhaus, Ron A. M. Fouchier, Sander Herfst

**Affiliations:** Viroscience Lab, Postgraduate School Molecular Medicine, Erasmus Medical Center, Rotterdam, The Netherlands; The Ohio State University, United States of America

## Abstract

Highly pathogenic avian influenza H5N1 viruses have devastated the poultry industry in many countries of the eastern hemisphere. Occasionally H5N1 viruses cross the species barrier and infect humans, sometimes with a severe clinical outcome. When this happens, there is a chance of reassortment between H5N1 and human influenza viruses. To assess the potential of H5N1 viruses to reassort with contemporary human influenza viruses (H1N1, H3N2 and pandemic H1N1), we used an *in vitro* selection method to generate reassortant viruses, that contained the H5 hemagglutinin gene, and that have a replication advantage *in vitro*. We found that the neuraminidase and matrix gene segments of human influenza viruses were preferentially selected by H5 viruses. However, these H5 reassortant viruses did not show a marked increase in replication in MDCK cells and human bronchial epithelial cells. In ferrets, inoculation with a mixture of H5N1-pandemic H1N1 reassortant viruses resulted in outgrowth of reassortant H5 viruses that had incorporated the neuraminidase and matrix gene segment of pandemic 2009 H1N1. This virus was not transmitted via aerosols or respiratory droplets to naïve recipient ferrets. Altogether, these data emphasize the potential of avian H5N1 viruses to reassort with contemporary human influenza viruses. The neuraminidase and matrix gene segments of human influenza viruses showed the highest genetic compatibility with HPAI H5N1 virus.

## Introduction

Since the late 1990s, highly pathogenic avian influenza (HPAI) viruses of the H5N1 subtype have devastated the poultry industry of numerous countries of the eastern hemisphere. After 2004, H5N1 has spread from Asia to Europe, Africa, and the Middle East, resulting in the killing or culling of hundreds of millions of domestic birds. Occasionally, HPAI H5N1 viruses cross the species barrier and infect humans, sometimes with a severe clinical outcome. This direct transmission of HPAI H5N1 virus to humans was first detected in 1997 [1] and has continued to be reported ever since [2]. Luckily, these viruses do not transmit efficiently between humans. However, they may gain the ability to spread efficiently among humans through either virus adaptation to the new host, genetic reassortment (ie genetic mixing of viruses) with contemporary human influenza viruses, or both [3]–[6]. Reassortment has proven to be an important mechanism for influenza viruses to evolve. The influenza pandemics of 1957, 1968 and 2009 were the result of reassortment events [7]–[9].

To date, reassortment of H5N1 viruses with human influenza viruses has not been detected in nature. However, co-infection of H5N1 and human viruses in humans or pigs may provide a new opportunity for reassortment and the subsequent emergence of viruses with pandemic potential. Therefore, it is important to investigate the genetic compatibility of the genes of potential parental strains.

In previous studies, reassortment of avian H5N1 and human H3N2 has been investigated extensively *in vivo* [10]–[12]. It was found that H5N1-H3N2 reassortment resulted in a more pathogenic H5 virus in mice [10], [12]. Furthermore, reassortment was found to occur readily *in vivo*, with a high probability in the ferret upper respiratory tract [11]. However, none of the tested H3N2-H5N1 reassortant viruses had gained the ability to be transmitted between ferrets.

Recent studies have also investigated the replication kinetics of many reassortant viruses between the 2009 pandemic H1N1 (pH1N1) and H5N1 influenza virus *in vitro* and *in vivo* [13]–[15]. Co-infection of cultured cells with pH1N1 and H5N1 showed that these two viruses have high genetic compatibility and that some of these viruses displayed better replication kinetics *in vitro* [13]. In addition, increased pathogenicity was observed for a reassortant pH1N1 containing an H5N1 HA in mice [15].

Avian H5N1 and human influenza viruses display different replication characteristics in primary cell cultures. Avian influenza viruses can infect human airway epithelium cells, although replication may be limited compared to human influenza viruses because of a nonoptimal cellular tropism [16]. This system offers an alternative to study virus and/or host properties required for adaptation or reassortment of influenza viruses. It was studied that co-infection of cells with viruses carrying HA of avian and human influenza viruses take place when the cells provide both receptors [17].

Here, we investigated the ability of HPAI H5N1 and contemporary human H3N2, H1N1 and pH1N1 influenza viruses to reassort, by means of an *in vitro* selection method using reverse genetics and serial passaging under limited dilution conditions as described before [18]. In contrast to double infection with 2 viruses, this method allows the production of gene segments at approximately similar copy numbers upon transfection, after which *in vitro* or *in ovo* viruses may differ in replication capacity. In addition, avian H5N1 outcompetes human influenza viruses in co-infection experiments [13]. In this way, a bias towards reassortants produced is thus prevented. The reassortants that were selected during this *in vitro* selection experiment were subsequently evaluated for replication kinetics in MDCK cells and normal human bronchial epithelial (NHBE) cultures. In addition, to study wether reassortants were produced with a replication advantage over the parental viruses *in vivo*, ferrets were inoculated with a mixture of reassortant viruses between pH1N1 and H5N1. The genetic composition of these reassortant mixtures were followed over time. Simultaneously, the transmissibility of these viruses was evaluated in a ferret model via aerosols or respiratory droplets.

## Materials and Methods

### Ethics Statement

Animals were housed and experiments were conducted in strict compliance with European guidelines (EU directive on animal testing 86/609/EEC) and Dutch legislation (Experiments on Animal Act, 1997). All animal experiments were approved by the independent animal experimentation ethical review committee ‘stichting DEC consult’ (Erasmus MC permit number EUR 1621) and were performed under animal biosafety level 3+ conditions as described elsewhere [6]. Animal welfare was observed on a daily basis, and all animal handling was performed under light anesthesia using ketamine to minimize animal suffering. Influenza virus sero-negative 6 month old female ferrets (*Mustella putorius furo*), weighing 800–1000 g., were obtained from a commercial breeder. All experiments involving H5N1 transmission were conducted prior to the institution of the current moratorium.

### Cells and Viruses

Madin-Darby Canine kidney (MDCK) cells were cultured in EMEM (Cambrex, Heerhugowaard, the Netherlands) supplemented with 10% FCS, 100 IU/ml penicillin, 100 µg/ml streptomycin, 2 mM glutamine, 1.5 mg/ml sodiumbicarbonate (Cambrex), 20 mM Hepes (Cambrex), and 0.1 mM non-essential amino acids (MP Biomedicals Europe, Illkirch, France). 293T cells were cultured in DMEM (Cambrex) supplemented with 10% FCS, 100 IU/ml penicillin, 100 mg/ml streptomycin, 2 mM glutamine, 1 mM sodium pyruvate, and non-essential amino acids.

All eight gene segments of influenza virus isolates A/Netherlands/602/2009 (pH1N1), A/Netherlands/213/2003 (H3N2), A/Netherlands/26/2007 (sH1N1) and A/Indonesia/5/2005 (H5N1) were amplified by reverse transcription polymerase chain reaction, cloned in a modified version of the bidirectional reverse genetics plasmid pHW2000 [19], [20], and subsequently used to generate recombinant virus by reverse genetics as described elsewhere [19]. All *in vitro* selection experiments, growth curves and virus titrations were performed under ABSL3+ conditions.

### Generation of the Reassortant Viruses

Reverse genetics was used to generate mixtures of reassortant viruses in 293T cells by co-transfecting eight plasmids that encode the H5N1 virus genome together with seven plasmids encoding the pH1N1, H3N2 or sH1N1 virus genome. The HA gene of these human viruses was omitted to make sure that only viruses with the HA of H5N1 virus were generated, as described previously [18]. The 293T cell supernatants were subsequently passaged in quadruplicate under limiting dilution conditions by using ten-fold serial dilutions in MDCK cells three times to enable selective outgrowth of viruses with high *in vitro* replication rates. Next, the genome composition of these viruses was determined by Sanger sequencing using conserved primers targeting the noncoding regions of each gene segment. Reverse genetics was subsequently used to produce reassortant viruses with the different gene compositions as identified by sequencing.

### Virus Titrations on MDCK Cells

Virus titers in ferret nasal and throat swabs, or samples from replication curves were determined by end-point titration in MDCK cells. MDCK cells were inoculated with tenfold serial dilutions of each sample, washed one hour after inoculation with phosphate-buffered saline (PBS), and cultured in 200 µl of infection medium, consisting of EMEM supplemented with 100 U/ml penicillin, 100 µg/ml streptomycin, 2 mM glutamine, 1.5 mg/ml sodiumbicarbonate, 20 mM Hepes, non-essential amino acids, and 20 µg/ml trypsin (Cambrex). Three days after inoculation, the supernatants of inoculated cell cultures were tested for agglutinating activity using turkey erythrocytes as an indicator of virus replication in the cells. Infectious virus titers were calculated from 4 replicates by the method of Spearman-Karber [21].

### Replication Kinetics in MDCK Cells

Multicycle replication curves were generated by inoculating MDCK cells at a multiplicity of infection (MOI) of 0.01 50 percent tissue culture infectious doses (TCID_50_) per cell in duplicate. Supernatants were sampled at 6, 12, 24, and 48 hours after inoculation, and virus titers in these supernatants were determined by means of end-point titration in MDCK cells as described above.

### Culture of NHBE Cells

Normal human bronchial epithelial (NHBE) cells were obtained from Clonetics (Basel, Switzerland) and used at passage 3–4. Undifferentiated NHBE cells were grown on 30 µg/ml type I collagen-coated 75 cm^2^ flasks in serum-free bronchial epithelial cell basal medium (BEBM) supplemented with BEBM SingleQuots (Clonetics). At 60–80% confluency, cells were trypsinized and seeded at a cell density of 1×10^4^ viable cells onto type I collagen-coated 6.5 mm transwell inserts with 0.4 µm pore size (Corning, Amsterdam, The Netherlands). The growth medium consisted of a 1∶1 mixture of complete BEBM and DMEM, supplemented with 15 ng/ml retinoic acid (Sigma-Aldrich). The medium was replaced with fresh medium every other day until cells reached confluency. Subsequently, an air-liquid interface (ALI) was created by removing medium from the apical side to promote mucocilliary differentiation. The medium was refreshed basolaterally and the apical side was washed with Dulbecco’s PBS (DPBS, Lonza) at 37°C every other day. Well-differentiated (wd-) NHBE cells were inoculated with influenza virus, 21 days after ALI, at which stage beating cilia and mucus production were clearly detectable.

### wdNHBE Cell Characterization

wdNHBE cells on transwell filters were washed with PBS, fixed with 4% paraformaldehyde for 20 min at room temperature (RT) and subsequently washed with PBS 0.1% Tween and permeabilized with 1% Triton X-100 in PBS. After washing with PBS and blocking with PBS 5% BSA-0.1% Tween, ciliated cells were identified by staining with mouse monoclonal β-Tubulin antibody (KLINIPATH, Duiven, The Netherlands). Goblet cells were identified by mouse Mucin 5AC antibody (MUC5AC, ITK DIAGNOSTIC BV, Uithoorn, The Netherlands). The cultures were subsequently incubated with a secondary goat anti-mouse IgG (H+L) texas red labelled antibody (Alexa Fluor ® 594, Invitrogen) to visualize ciliated cells or goblet cells. In addition, cell cultures stained for the presence of ciliated cells were double stained to also visualize the tight junctions: cells were incubated with a ZO-1 N-Term antibody (Invitrogen), followed by incubation with a secondary swine anti-rabbit FITC labelled antibody (DAKO, Enschede, Netherlands). The transwell filters were cut off after staining, mounted on slides with Prolong Gold Mount (Vectashield, Peterborough, UK) and analysed using a fluorescence imaging microscope (Leica Microsystems).

### Lectin Staining of Differentiated NHBE Cells

The wdNHBE cultures were washed with DPBS to remove overlaying mucus and incubated for 1 hour at 4°C with biotin-labeled lectines *sambucus nigra* agglutinin (SNA; specific for sialic acid α2.6 Gal; 4 µg/ml; SANBIO BV, Uden, Netherlands) or *Maackia amurensis* agglutinin II (MAAII; specific for sialic acid α2.3 Gal; 20 µg/ml; BIO-CONNECT, Huissen, Netherlands) in Tris-buffered saline (TBS; pH 7.2) containing 1% BSA and 1 mM Mg^2+^, Ca^2+^, and Mn^2+^. Next, the cells were washed with TBS and fixed with 4% paraformaldehyde. After washing, the cultures were incubated for 1 hour at RT with streptavidin-horseradish peroxidise FITC-labeled conjugate (DAKO) in 1% BSA-TBS. Cultures were washed, permeabilized and stained for cilia as described above and analysed by confocal laser scanning microscopy using a LSM700 system fitted on an Axio Observer Z1 inverted microscope (Zeiss). Images were generated using Zen software.

### Replication Kinetics in Differentiated NHBE Cells

The wdNHBE cells were washed with DPBS to remove overlaying mucus and duplicates were inoculated via the apical side, with virus of interest at a MOI of 0.02 in 100 µl. After one hour of incubation at 37°C the inoculum was removed, cells were washed three times with DPBS and once with growth medium. The last wash step with growth medium was collected for virus titration as time point t = 0. At 6, 12, 24, and 48 hours after inoculation, 100 µl of growth medium was added to the apical side of the trans well of each culture to collect virus samples. After 10 min of incubation at 37°C, the medium was collected and stored at −80°C for virus titration in MDCK cells.

### Ferret Transmission Experiment

The ferret model to test for aerosol or respiratory droplet transmission was described previously [22]. In the transmission experiment, two influenza virus sero-negative female ferrets were individually housed in transmission cages and inoculated intranasally, divided over both nostrils (2×250 µl), with 10^7.3^ TCID_50_ of MDCK passage 1 (MDCKP1) of the H5N1-pH1N1 reassortant virus mixture (eight H5N1 and seven pH1N1 plasmids, without the pH1N1 HA, were transfected in 293T cells and supernatant was subsequently passaged on MDCK cells). At 1 day post inoculation (dpi), naïve recipient ferrets were individually placed in a transmission cage adjacent to a donor ferret. The animals were separated by two stainless steel grids to allow airflow from the donor to the recipient ferret but to prevent direct contact- and fomite transmission. Nasal and throat swabs were collected at 1, 3, 5 and 7 dpi from donor ferrets and at 1, 3, 5 and 7 days post exposure (dpe) from the recipient animals. Donor ferrets were euthanized at 7 dpi and recipient ferrets at 7 dpe. Virus titers were determined in collected swabs by means of end-point titration in MDCK cells.

### Analysis of Viral Genome Composition in Ferrets by Pyrosequencing

Pyrosequencing, a method to detect single nucleotide polymorphisms (SNP) [23], was used to determine the exact proportion of H5N1 and pH1N1 gene segments in the donor ferrets inoculated with the MDCK P1 H5N1-pH1N1 reassortant mixture. RNA was isolated from the throat swabs collected at 1, 3, 5 and 7 days dpi. After cDNA synthesis, conserved primers were used for each gene segment to amplify a small PCR product of approximately 100 bp (Table 1). These fragments were next sequenced using the Pyromark Q24 pyrosequencing platform (Qiagen, Venlo, The Netherlands) with a specific sequence primer (Table 1).

**Table 1:** Primers used for pyrosequencing.

Primers	Sequence (5′-3′)
Forward PB2 H5N1/H1N1	GCAGGTCAAATATATTCAATATGG
Reverse PB2 H5N1/H1N1	GATTATGGCCATATGGTCCAC * biotin
Sequence primer	TCAATATGGAGAGAATAAAA
Forward PB1 H5N1/H1N1	CAGGATACACCATGGACACAGT
Reverse PB1 H5N1/H1N1	CCTCAGGTAGTGGTCCATCAATC* biotin
Sequence primer	ATACACCATGGACACAGT
Forward PA H5N1/H1N1	GTGCGACAATGCTTCAATCCA
Reverse PA H5N1/H1N1	GTGTGCATATTGCAGCAAA * biotin
Sequence primer	CAATGCTTCAATCCAA
Forward NP H5N1/H1N1	GAGCTCTCGGACGAAAAGG
Reverse NP H5N1/H1N1	CTCTGCATTGTCTCCGAAGAA * biotin
Sequence primer	TGCCTTCCTTTGACAT
Forward NA H5N1/H1N1	GGCATAATAACAGACACTATCAAG
Reverse NA H5N1/H1N1	CCATTACTTGGTCCATC * biotin
Sequence primer	GACACTATCAAGAGTTGGAG
Forward MA H5N1/H1N1	GAGTCTTCTAACCGAGGTCGAAAC * biotin
Reverse MA H5N1/H1N1	GTGTTCTTTCCTGCAAAGAC
Sequence primer	GCCTGACGGGATGATA
Forward NS H5N1/H1N1	AGGGTGACAAAAACATAATGGA
Reverse NS H5N1/H1N1	CAAGGAATGGGGCATCACCC * biotin
Sequence primer	GTGACAAAAACATAATGGA

## Results

### *In vitro* Selection of H5 Reassortant Viruses

Reverse genetics was used to generate mixtures of reassortant viruses in 293T cells, by co-transfecting eight plasmids that encode the H5N1 virus genome together with seven plasmids encoding the pH1N1, H3N2 or sH1N1 virus genome, omitting their HA gene. The 293T cell supernatants were passaged, in quadruplicate, under limiting dilution conditions to allow selective outgrowth of viruses with high *in vitro* replication rates. After three passages, the genome composition of these viruses was determined by sequencing (Table 2). The predominant virus population was identified for almost all passaged virus mixtures by sanger sequencing, with minor virus variants representing less than 20% of the virus mixture (estimated detection limit for sanger sequencing). Point mutations were not observed in the proportion of the genome analyzed. Upon H5N1-pH1N1 transfection and passaging of the virus mixtures, wild type (wt) H5N1 was recovered in one attempt and H5N1-pH1N1-reassortants in three attempts (Table 2). These reassortants incorporated the pH1N1 matrix (M) gene (H5-pH1_M_), or the pH1N1 M, neuraminidase (NA) and non-structural (NS) genes (H5-pH1_NA,M,NS_), or the pH1N1 M, NA, NS and polymerase complex genes PB2, PB1 and PA (H5-pH1_NA,M,NS,PB2,PB1,PA_).

**Table 2:** Predominant virus genome composition upon *in vitro* selection of mixtures of H5N1-pH1N1, H5N1-H3N2 and H5N1-sH1N1 reassortants.

Replicates	PB2	PB1	PA	HA	NP	NA	M	NS
**H5-pH1 1**	H5	H5	H5	*H5*	H5	H5	**pH1**	H5
**H5-pH1 2**	**pH1**	**pH1**	**pH1**	*H5*	H5	**pH1**	**pH1**	**pH1**
**H5-pH1 3**	H5	H5	H5	*H5*	H5	H5	H5	H5
**H5-pH1 4**	H5	H5	H5	*H5*	H5	**pH1**	**pH1**	H5**/pH1** *
**H5-H3 1**	H5	H5	H5	*H5*	H5	**H3**	H5	H5
**H5-H3 2**	H5	H5	H5	*H5*	H5	H5	H5	H5
**H5-H3 3**	H5	H5	H5	*H5*	H5	**H3**	H5/**H3** **	H5
**H5-H3 4**	H5	H5	H5	*H5*	H5	**H3**	H5	H5
**H5-sH1 1**	H5	H5	H5	*H5*	H5	H5	**sH1**	H5
**H5-sH1 2**	H5	H5	H5	*H5*	H5	**sH1**	**sH1**	H5
**H5-sH1 3**	H5	H5	H5	*H5*	H5	**sH1**	H5	H5
**H5-sH1 4**	H5	H5	H5	*H5*	H5	H5	H5	H5

* A 25/75 population of NS H5 and pH1 was detectable.

** A 50/50 population of M H5 and H3 was detectable.

Upon H5N1-H3N2 transfection and passaging, wtH5N1 was recovered in one attempt, whereas three reassortant viruses were recovered that had the NA gene of H3N2 (H5-H3_NA_). In one of the reassortant viruses that contained the NA gene of H3N2, a mixed population was present for the M gene (approximately 50% H5N1 and 50% H3N2; H5-H3_NA_,_M_) (Table 2).

Four different genome compositions were identified upon H5N1-sH1N1 transfection and passaging: wtH5N1 was recovered as well as three reassortant viruses containing the sH1N1 M or NA or M and NA genes (H5-H1_NA_, H5-H1_M_ and H5-H1_NA,M_) (Table 2).

### *In vitro* Characterization of H5 Reassortant Viruses in MDCK Cells

**Figure 1:**
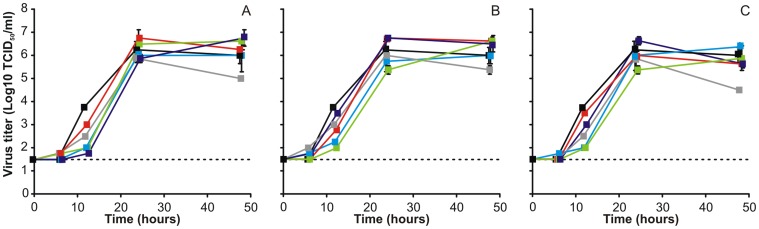
Replication kinetics of H5N1-pH1N1, H5N1-H3N2 and H5N1-sH1N1 reassortant viruses in MDCK cells. MDCK cells were inoculated with 0.01 TCID50/cell of H5N1 (black), H5 reassortant viruses harboring the NA (grey), M (red), NS (purple), NA and M (blue) and NA, M and NS (green) of pH1N1 (A), H3N2 (B) or sH1N1 (C) and supernatant samples were harvested 6, 12, 24, and 48 h later. Geometric mean titers were calculated from two independent experiments, error bars indicate standard deviations. The lower limit of detection is indicated by the dotted line.

The data obtained from the *in vitro* selection experiments suggest that the NA and M genes of all three tested human influenza viruses as well as the NS gene of pH1N1 frequently substituted their H5N1 counterparts. Therefore, reverse genetics was used to generate H5 reassortants that contained one (NA, M or NS), two (NA and M), or three (NA, M and NS) genes of pH1N1, H3N2 or sH1N1 (Table 3) and their replication kinetics was subsequently evaluated in MDCK cells. MDCK cells were inoculated with reassortant viruses at an MOI of 0.01, after which the supernatants were harvested at fixed time points and virus titers were determined in MDCK cells. In general, H5 reassortant viruses containing gene segments of pH1N1 showed similar virus titers compared to wtH5N1 (Fig. 1A), although H5-pH1_M_ and H5-pH1_NA,M,NS_ reassortant viruses were produced at a slightly higher level at 24 and 48 hours post-inoculation (pi), whereas H5-pH1_NA_ had a lower replication rate compared to wtH5N1. The virus replication of most H5N1-H3N2 reassortant viruses was similar to that of wtH5N1 in MDCK cells (Fig. 1B), however H5-H3_M_ and H5-H3_NS_ reassortant viruses had slightly increased virus titers at 24 and 48 hours pi, whereas H5-H3_NA_ had a lower virus titer at 48 hours pi. Almost all H5 reassortant viruses with sH1N1 gene segments replicated to similar virus titers compared to wtH5N1, although H5-sH1_NA_ had a lower virus titer at 48 hours pi, compared to wtH5N1. Although the differences in replication kinetics between the wtH5N1 and the H5 reassortant viruses were rather small, it seemed that incorporation of any of the three NA’s attenuated virus replication at 48 hours pi.

**Table 3:** H5 Reassortant influenza viruses rescued via reverse genetics.

Reassortant virus	PB2	PB1	PA	HA	NP	NA	M	NS
**H5N1**	H5	H5	H5	*H5*	H5	H5	H5	H5
**H5-pH1_NA_**	H5	H5	H5	*H5*	H5	**pH1**	H5	H5
**H5-pH1_M_**	H5	H5	H5	*H5*	H5	H5	**pH1**	H5
**H5-pH1_NS_**	H5	H5	H5	*H5*	H5	H5	H5	**pH1**
**H5-pH1_NA,M_**	H5	H5	H5	*H5*	H5	**pH1**	**pH1**	H5
**H5-pH1_NA,M,NS_**	H5	H5	H5	*H5*	H5	**pH1**	**pH1**	**pH1**
**H5-H3_NA_**	H5	H5	H5	*H5*	H5	**H3**	H5	H5
**H5-H3_M_**	H5	H5	H5	*H5*	H5	H5	**H3**	H5
**H5-H3_NS_**	H5	H5	H5	*H5*	H5	H5	H5	**H3**
**H5-H3_NA,M_**	H5	H5	H5	*H5*	H5	**H3**	**H3**	H5
**H5-H3_NA,M,NS_**	H5	H5	H5	*H5*	H5	**H3**	**H3**	**H3**
**H5-sH1_NA_**	H5	H5	H5	*H5*	H5	**sH1**	H5	H5
**H5-sH1_M_**	H5	H5	H5	*H5*	H5	H5	**sH1**	H5
**H5-sH1_NS_**	H5	H5	H5	*H5*	H5	H5	H5	**sH1**
**H5-sH1_NA,M_**	H5	H5	H5	*H5*	H5	**sH1**	**sH1**	H5
**H5-sH1_NA,M,NS_**	H5	H5	H5	*H5*	H5	**sH1**	**sH1**	**sH1**

### *In vitro* Characterization of H5 Reassortant Viruses in wdHBE Cells

Influenza viruses infect cells of the respiratory tract of humans. Therefore, we evaluated the replication kinetics of the reassortant viruses in wdNHBE cells.

To characterize the wdNHBE cells, immunohistochemistry was used to identify ciliated cells, mucus-producing goblet cells and tight junctions. Cultures were also double stained for ciliated cells and tight junctions. For further characterization, the sialic acid (SA) receptor distribution on wdNHBE cells was determined by lectin histochemistry using MAL-II which recognizes the α2.3-linked SA and SNA which recognizes α2.6-linked SA, the receptors for avian and human influenza viruses respectively. This data is in agreement with the general pattern of SA receptor distribution on wdNHBE cells cultured *in vitro*: α2.3-linked SA receptors are expressed predominantly on ciliated cells and to a lesser extent on nonciliated cells and α2.6-linked SA receptors are expressed mainly on nonciliated cells and to a lesser extent on ciliated cells [16].

**Figure 2:**
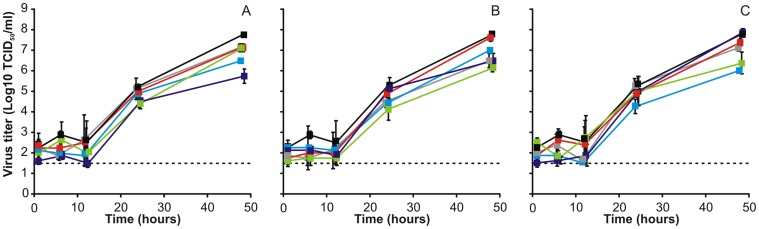
Replication kinetics of H5N1-pH1N1, H5N1-H3N2 and H5N1-sH1N1 reassortant viruses in wdNHBE cells. wdNHBE cells were inoculated with 0.02 TCID50/cell of H5N1 (black), H5N1 reassortant viruses consisting of the NA (grey), M (red), NS (purple), NA and M (blue) and NA, M and NS (green) of pH1N1 (A), H3N2 (B) or sH1N1 (C) and samples were harvested 1, 6, 12, 24, and 48 h later. Geometric mean titers were calculated from two independent experiments, error bars indicate standard deviations. The lower limit of detection is indicated by the dotted line.

To study the replication kinetics of the H5 reassortant viruses, wdNHBE cells were inoculated with an MOI of 0.02. Growth medium was added for ten minutes to harvest virus at fixed time points. None of the H5N1-pH1N1 (Fig. 2A), H5N1-H3N2 (Fig. 2B) and H5N1-sH1N1 (Fig. 2C) reassortant viruses replicated to higher virus titers then wtH5N1 in wdNHBE cells. Some H5 reassortant viruses (H5-pH1_NS_, H5-H3_NA,M,NS_ and H5-sH1_NA,M_) even displayed virus titers at 48 hours p.i. that were >1.5 log10 TCID_50_/ml lower compared to the wtH5N1 virus titer.

### H5N1-pH1N1 Reassortant Viruses in Ferrets

**Figure 3:**
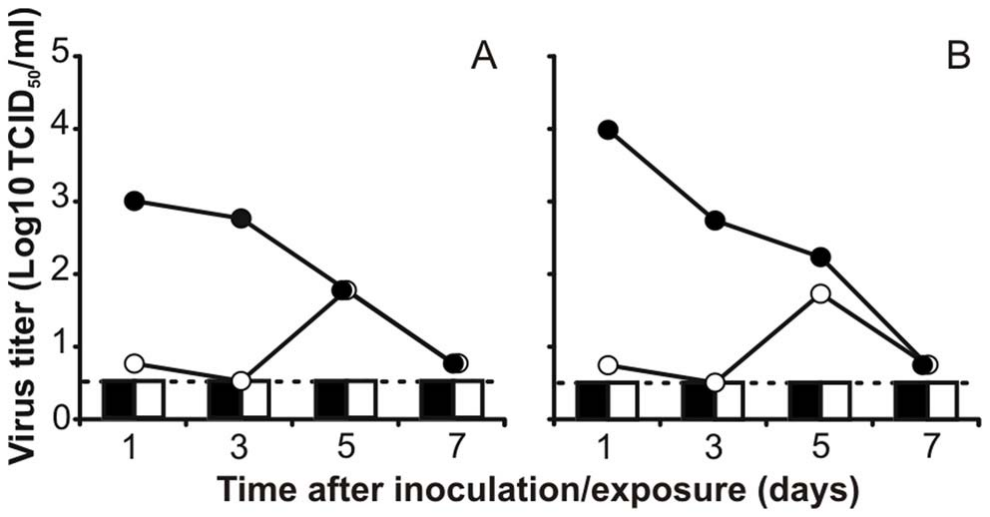
Replication and transmission of H5N1-pH1N1 reassortant virus in ferrets. Two ferrets were inoculated intranasally with 10^7.3^ TCID_50_ of the MDCKP1 H5N1-pH1N1 reassortant virus mixture and subsequently housed individually in transmission cages (A, B). A naïve recipient ferret was added to a cage adjacent to each transmission cage at 1dpi (A, B). Virus titers in throat (black) and nose swabs (white) of the donor ferrets (lines) and recipient ferrets (bars) were determined by endpoint titration in MDCK cells. The lower limit of detection is indicated by the dotted line.

The ferret model was used to select for H5N1-pH1N1 reassortant viruses with highest replication *in vivo*, and to evaluate the ability of this virus to be transmitted via aerosols or respiratory droplets. Two ferrets were inoculated intranasally with 10^7.3^ TCID_50_ of the MDCKP1 H5N1-pH1N1 reassortant virus mixture. A recipient ferret was placed in a transmission cage adjacent to each donor ferret one day later. Throat and nose swabs were collected at 1, 3, 5 and 7 dpi and dpe. Virus shedding peaked in the inoculated animals at 1 dpi, with virus titers up to 10^4^ TCID_50_/ml in throat swabs (Fig. 3), and this shedding continued until 7 dpi. Overall, the amount and duration of virus shedding of ferrets inoculated with the reassortant H5N1-pH1N1 mixture was lower compared to those of wild type H5N1-inoculated ferrets in our previous experiment [22]. None of the viruses in the H5N1-pH1N1 reassortant mixture was transmitted to the recipient ferrets via the aerosol or respiratory droplet route, since no virus could be detected in the throat and nose swabs collected from the recipient ferrets (Fig. 3).

**Figure 4:**
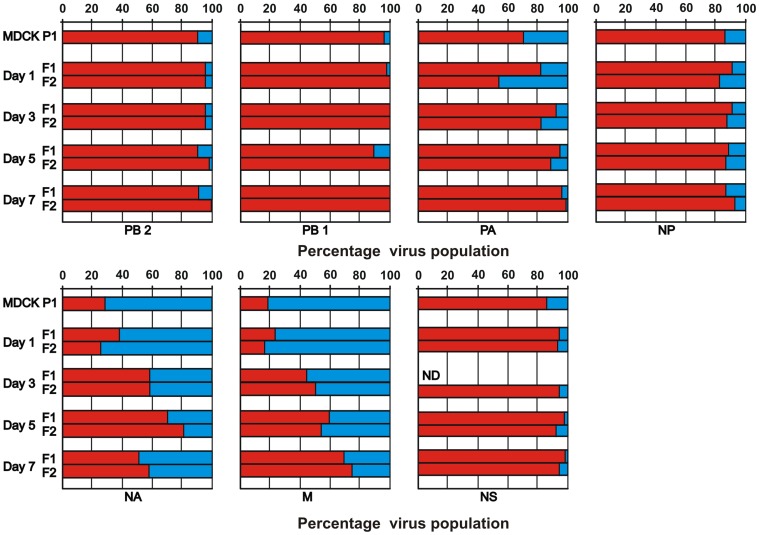
Virus composition in ferrets inoculated with H5N1-pH1N1 reassortant virus during the course of infection. Two ferrets were inoculated intranasally with 10^7.3^ TCID_50_ of the MDCKP1 H5N1-pH1N1 reassortant virus mixture. At day 1, 3, 5 and 7 dpi throat swabs were collected from both ferrets (F1 and F2). In these throat samples, as well as the MDCKP1 inoculum, the ratio of the H5N1 (red) and pH1N1 (blue) gene segments (PB2, PB1, PA, NP, NA, M and NS) was determined by pyrosequencing. ND: no detection of viral gene segments by PCR.

The genetic composition of the H5N1-pH1N1 reassortant viruses in throat swabs collected from the donor ferrets as well as in the MDCKP1 virus stock that was used as inoculum, was analyzed using pyrosequencing. In the MDCKP1 virus stock, the vast majority of the PB2, PB1, PA, NP and NS genes was derived from H5N1, and these H5 genes remained dominant in both ferrets until 7 dpi: 95±5%, 100±0%, 98±2%, 90±4% and 96±3% respectively (Fig. 4). In contrast, the NA and M gene segments of pH1N1 virus origin were dominant in the inoculum. In addition, these pH1N1 genes were still present in the reassortant mixture collected from ferrets at 7 dpi, although the proportion of these genes had decreased during the course of infection to 46±4% and 28±4% for NA and M respectively (Fig. 4).

## Discussion

Genetic reassortment is an important mechanism in the evolution of influenza viruses yielding strains with novel genetic and phenotypic traits. At least two human influenza pandemics in the last century were linked to lineages where circulating human influenza viruses reassorted with influenza genes of non-human (probably avian) origin [9]. To study the potential of reassortment between the HPAI H5N1 virus and contemporary human influenza viruses (pH1N1, H3N2 and sH1N1), we used an *in vitro* selection method to identify the reassortant viruses that are most likely to emerge. The HA of the contemporary human influenza viruses was omitted in these experiments to make sure that only viruses containing an H5 HA were generated. In this way, a mixture of up to 128 (2^7^) possible different reassortant viruses was generated that was subsequently passaged in MDCK cells under limiting dilution conditions to allow selective outgrowth of viruses with high *in vitro* replication rates. It should be noted that virus replication in MDCK cells may not reflect natural selection of reassortant viruses in humans. However, we have previously shown that with this method, reassortant viruses with enhanced pathogenicity in ferrets could be identified, thereby emphasizing the usefulness of this method [18].

Analysis of the genetic composition of the viruses that were obtained using the *in vitro* selection method in the present study, showed that wtH5N1 virus was recovered in 1 out of 4 attempts in all three H5N1-human influenza genome mixing experiments. In all other attempts, the NA and M genes of pH1N1, H3N2 and sH1N1 as well as the NS gene of pH1N1 were selected by HPAI H5 virus, after replication in mammalian cells (Table 2).

In addition, the ability of H5N1 and pH1N1 influenza viruses to reassort has recently been investigated by others. In this study, MDCK cells were coinfected with these two viruses, resulting in the selection of similar reassortant viruses, harboring the NA, M, and NS gene segments of pH1N1 [13]. In another study, ferrets were co-infected with avian H5N1 and human H3N2 resulting in reassortant viruses that had also incorporated the NA, M and NS gene segments of a human H3N2 virus [11]. Thus, our transfection based approach yielded similar data as with other methods.

When we studied the replication kinetics of the H5 reassortant viruses that were detected after *in vitro* selection in MDCK cells, we found that only a few H5 reassortant viruses had a slightly increased replication capacity compared to the wt H5N1 virus (Fig. 1). This effect could for the most part be attributed to the M gene segment of pH1N1 and H3N2. In contrast, when the NA of H5N1 was exchanged by the NA of one of the three human influenza viruses, the replication kinetics were slower compared to the wtH5N1 virus, which is in agreement with previous findings when H5N1-H3N2 reassortment was studied [11].

In wdNHBE cells, HPAI H5N1 replicates to lower titers compared to pH1N1, H3N2 and sH1N1 (data not shown and [24]). However, when the replication capacity of H5 reassortant viruses was investigated, no increased replication was demonstrated for any of the H5 reassortants, when compared to wtH5N1. It is possible that this poor replication of H5N1 virus compared to the human influenza viruses is the result of the avian receptor specificity of H5N1. Modification of the receptor binding preference of H5N1 to the human type receptors may result in increased replication in wdNHBE cells, and may to some extent compensate for the need of reassortment with human influenza viruses.

The data obtained with the *in vitro* selection experiments showed that especially the M and NA gene segments from human influenza viruses are preferentially selected by avian H5N1. However when these reassortant viruses were investigated *in vitro* none of the reassortant H5 viruses had an apparent increase in replication capacity compared to wtH5N1. This observation may explain why wtH5N1 was also recovered in all three *in vitro* selection experiments.

To study if reassortment between H5N1 and pH1N1 can be beneficial for virus replication in mammals, ferrets were inoculated with a mixture of reassortant viruses (MDCKP1 virus stock) and the virus composition was determined at different time points during the course of infection using pyrosequencing. In the inoculum, the H5 polymerase complex genes PB2, PB1 and PA, as well as the H5N1 NP and NS genes were predominant, with only a small percentage of these genes being derived from pH1N1. Although the H5N1 polymerase genes did not contain the well-known mutations in PB2 (E627K or D701N) that have been shown to be required for optimal replication of avian influenza viruses in mammals [25], [26], the proportion of the pH1N1 polymerase complex genes in the virus mixture remained low during the course of infection. In contrast, the proportion of the pH1N1 NA and M genes in the MDCKP1 virus mixture were higher than those of the H5N1 counterparts, but this proportion decreased slowly over time. It should be noted that the assessment of the preference of reassortant H5 viruses for H5N1 or pH1N1 gene segments would be more reliable if all gene segments would be present in equal copy numbers in the inoculum. Unfortunately, the generation of reassortants by transfecting 293T cells with plasmids harboring the different gene segments apparently resulted in a biased virus population with already a strong preferred use of some of the H5 genes. However, gene segments that represented only a small proportion of the population (like the pH1N1 polymerase genes) should have increased in number if there would have been an apparent selective advantage for the viruses.

In the same ferret experiment, we investigated whether an airborne-transmissible H5N1 virus was present in the reassortant virus mixture. However, no aerosol or respiratory droplet transmission was detected, since no virus could be detected in respiratory samples collected from the naïve recipient ferrets. This may be the result of the low amount of virus that was shed by the donor ferrets, combined with the receptor binding preference of the H5N1 HA for α2,3-linked SA that are absent in the upper respiratory tract of ferrets. This switch in receptor preference was recently shown to be crucial for airborne transmission of H5N1 viruses [3], [6]. However, H5N1 viruses that were only mutated to acquire a preference for human α2,6-linked SA receptors were not transmitted between ferrets, suggesting that additional genetic changes are needed [6], [27]. Recently Imai et al. showed that in addition to receptor binding preference for α2,6-linked SAs, two additional mutations in HA are required to confer a H5N1-pH1N1 reassortant, carrying the H5 HA and the other genes from pH1N1, airborne-transmissible between ferrets [3]. Moreover, recently we discovered that a fully avian H5N1 virus, with a preference for for human α2,6-linked SA receptors, can acquire the ability to be transmitted between ferrets without the need for reassortment [6].

Although none of the reassortant viruses identified and evaluated in our study have an evident replication advantage over their parental viruses, the generated reassortant viruses were also not found to be severely attenuated. Given that only a few mutations are necessary to confer airborne transmission of a H5N1-pH1N1 reassortant between ferrets, the emergence of reassortant viruses between human and avian influenza viruses but also between human and porcine influenza viruses should be monitored carefully.
